# Professional Autonomy, Professional Quality of Life, and Moral Courage Among Emergency Nurses: A Cross‐Sectional Study in Northern Iran

**DOI:** 10.1155/jonm/9169873

**Published:** 2026-10-25

**Authors:** Vida Shafipour, Mohammad Javad Ahmadzadeh-zeidi, Tahereh Yaghoubi, Somayeh Ghorbani, Seyednouraddin Mousavinasab

**Affiliations:** ^1^ Traditional and Complementary Medicine Research Center Addiction Institute Mazandaran University of Medical Sciences Sari Iran mazums.ac.ir; ^2^ Department of Nursing Nasibeh Nursing and Midwifery Faculty Mazandaran University of Medical Sciences Sari Iran mazums.ac.ir; ^3^ Department of Medical-Surgical Nursing Faculty of Nursing and Midwifery Tehran University of Medical Sciences Tehran Iran tums.ac.ir; ^4^ Department of Emergency Nursing Nasibeh Faculty of Nursing and Midwifery Mazandaran University of Medical Sciences Sari Iran mazums.ac.ir; ^5^ Department of Biostatistics Faculty of Health Mazandaran University of Medical Sciences Sari Iran mazums.ac.ir

**Keywords:** compassion satisfaction, emergency nurses, moral courage, professional autonomy, professional quality of life

## Abstract

**Background and Aim:**

Emergency nurses operate in highly demanding environments where professional autonomy is crucial for delivering safe and effective patient care. While Moral Courage and Professional Quality of Life (ProQoL) have both been linked to professional outcomes, their relationships with autonomy remain insufficiently understood in high‐acuity emergency settings. This study aimed to examine the relationships among professional autonomy, moral courage, and ProQoL among emergency nurses in northern Iran.

**Materials and Methods:**

This multicenter cross‐sectional study included 180 emergency nurses from 10 teaching hospitals in northern Iran in 2024. Participants were selected using proportionate stratified systematic random sampling. Autonomy, Moral Courage, and ProQoL were assessed using validated questionnaires. Data analysis employed descriptive statistics, Spearman’s correlation, independent *t*‐tests, one‐way ANOVA, Mann–Whitney *U* tests, Kruskal–Wallis tests, and multiple linear regression in SPSS Version 27 (*p* < 0.05).

**Results:**

The mean age of participants was 33.18 ± 7.35 years, with 55.0% being female. Emergency nurses reported high autonomy, moderate levels of moral courage, and moderate levels across all ProQoL dimensions. Autonomy was positively correlated with moral courage (*ρ* = 0.57, *p* < 0.001) and compassion satisfaction (*ρ* = 0.31, *p* < 0.001). Moral courage was also associated with compassion satisfaction (*ρ* = 0.31), burnout (*ρ* = 0.40), and secondary traumatic stress (*ρ* = 0.30) (*p* < 0.001). In multiple regression analysis, endurance of threats (*β* = 0.32, *p* = 0.001), multiple values (*β* = 0.26, *p* = 0.002), moral agency (*β* = 0.19, *p* = 0.004), and compassion satisfaction (*β* = 0.15, *p* = 0.032) were significant positive predictors of professional autonomy, explaining 39% of its variance (*R* ^2^ = 0.39, *p* < 0.001).

**Conclusion:**

Professional autonomy showed a stronger association with moral courage than with ProQoL, while compassion satisfaction was independently associated with greater autonomy.

**Implications for Nursing Management:**

Nurse managers should strengthen ethical practice, nurse empowerment, and supportive practice environments to create conditions that facilitate autonomous nursing practice.

## 1. Introduction

Emergency departments represent one of the most demanding clinical environments within healthcare systems [1]. Emergency nurses are continuously exposed to high patient acuity, unpredictable situations, traumatic events, ethical conflicts, and time‐sensitive decision‐making [2, 3]. These conditions substantially increase occupational demands and may threaten nurses’ ability to function effectively and independently in clinical practice [1]. Consequently, strengthening autonomy has become an important priority for healthcare organizations.

Professional autonomy, a hallmark of nursing professionalism, refers to nurses’ authority and ability to make independent decisions based on professional knowledge, competencies, and ethical responsibilities [4, 5]. Higher autonomy has been associated with improved quality of care, stronger professional identity, greater job satisfaction, and workforce retention, whereas limited autonomy contributes to moral distress, occupational stress, and turnover intentions [6–8]. Despite its importance, nurses generally report moderate levels of autonomy, and hierarchical and physician‐centered structures continue to restrict autonomous practice in Iran [9–11].

The Job Demands—Resources (JD‐R) model offers a valuable framework for comprehending professional functioning. This model posits that excessive job demands deplete physical and psychological resources, while personal and work‐related resources foster positive outcomes [12]. Consequently, autonomy can be seen as a crucial outcome supported by sufficient psychological and ethical resources.

One of the most important psychological resources in nursing is Professional Quality of Life (ProQoL), encompassing the positive and negative consequences of caring for individuals experiencing illness and suffering [13]. Stamm’s model defines ProQoL through three components: compassion satisfaction, burnout, and secondary traumatic stress. Compassion satisfaction represents positive experiences derived from helping others, whereas burnout and secondary traumatic stress represent the detrimental psychological effects of prolonged occupational stress and repeated exposure to patients’ suffering [14, 15].

Poor ProQoL has been associated with emotional exhaustion, impaired patient‐centered care, medical errors, absenteeism, and turnover intention, whereas higher ProQoL contributes to better functioning and organizational commitment [14, 16, 17]. In emergency settings, where nurses are repeatedly exposed to traumatic experiences and intense emotional demands, maintaining adequate ProQoL is particularly important [18]. Although international evidence suggests moderate levels of ProQoOL, Iranian studies have reported considerable variability, especially in highly demanding settings [15, 19]. Recent evidence among emergency nurses further emphasizes the importance of psychological resources in sustaining professional well‐being [20].

However, psychological well‐being alone may not be sufficient for effective professional practice. Emergency nurses frequently encounter ethical challenges involving end‐of‐life decisions, patient advocacy, resource allocation, medical errors, and interprofessional conflicts [21, 22]. Responding appropriately to these situations requires not only ethical awareness but also the willingness to act according to professional values despite potential risks and adverse consequences [23]. This capability is reflected in the concept of moral courage.

Moral courage is the ability to recognize ethical obligations and take morally appropriate actions despite fear or potential negative consequences [24, 25]. Sekerka’s framework identifies its components as moral agency, multiple values, endurance of threat, moral goals, and going beyond compliance [23]. In clinical nursing, moral courage empowers nurses to advocate for patients, challenge unsafe practices, uphold ethical principles, and translate moral judgments into ethical actions [26, 27].

Within the JD‐R framework, moral courage is considered a crucial personal resource that facilitates adaptive responses to ethical challenges. Greater moral courage is linked to ethical decision‐making, reduced moral distress, and safer care. Conversely, insufficient moral courage can contribute to burnout and compromised performance [24, 26, 28]. Evidence suggests that nurses globally, including those in Iran, generally report moderate levels of moral courage [28–30].

Although ProQoL and moral courage have been extensively studied, they have largely been examined in isolation. Prior research has primarily explored the links between ProQoL and occupational outcomes, and between moral courage and ethical competence or moral distress. Investigations into professional autonomy, meanwhile, have predominantly focused on organizational factors. Although emerging evidence has begun to examine overlapping relationships among these constructs, studies simultaneously addressing professional autonomy, ProQoL, and moral courage in emergency nursing remain limited, particularly within the Iranian context.

This gap is particularly relevant to emergency nursing, where high job demands and ethically complex situations require both autonomous decision‐making and sustained psychological and ethical resources. Examining the interrelationships among ProQoL, moral courage, and professional autonomy may, therefore, provide a more integrated understanding of factors associated with professional functioning in this setting. Accordingly, this study aimed to investigate the relationships among professional autonomy, ProQoL, and moral courage among emergency nurses in northern Iran.

## 2. Methods

### 2.1. Study Design and Participants

This multicenter cross‐sectional correlational study examined the relationships among professional autonomy, ProQoL, and moral courage among emergency nurses. The study was conducted in 2024 across all 10 teaching hospitals affiliated with Mazandaran University of Medical Sciences in northern Iran that met the study eligibility criteria. All eligible emergency departments within these hospitals were included, and eligible nurses were selected using systematic random sampling based on the predefined inclusion criteria. Eligible participants were full‐time nurses with at least a bachelor’s degree in nursing and ≥ 1 year of clinical experience in an emergency department. Questionnaires with > 10% missing responses were excluded.

The target population comprised 330 eligible emergency nurses identified from the provincial nursing office registry. The minimum sample size was calculated using Cochran’s formula for finite populations, assuming a 95% confidence level, a 5% margin of error, and maximum variability (*p* = *q* = 0.50), yielding a required sample of 177 nurses. To allow for potential incomplete responses, 200 questionnaires were distributed.

(1) n=Nz2pqNd2+z2pqn=3300.50.5×1.962××3300.50.5×0.052+1.962××=177.

A stratified systematic random sampling approach was used, with hospital as the stratification variable. The required sample from each hospital was determined proportionally to the number of eligible emergency nurses employed at that hospital. Subsequently, a complete sampling frame of eligible nurses was prepared for each hospital, and participants were selected systematically using the sampling interval (*K* = *N*/*n*), following a random starting point. Thus, nurses were independently selected from each hospital according to its allocated sample size, ensuring proportional representation across hospitals. Of the 200 distributed questionnaires, 180 were complete and eligible for analysis, yielding a 90% response rate and representing 54.5% of the target population.

### 2.2. Measures

Data for this study were collected using previously published and validated instruments: the Stamm’s ProQoL Scale [31], the Sekerka Moral Courage Scale (MCS) [32], and the Professional Autonomy Scale (Dempster Practice Behavior Scale [DPBS]) [33]. These questionnaires have been widely used in nursing research, and English versions are cited in the references.

Before initiating the study, permission was obtained from the developers of localized versions of the questionnaires. Data for this research were collected using a self‐report method through the following questionnaires:

A demographic and occupational questionnaire was used to collect information such as age, marital status, education level, employment status, work shift, work experience, and monthly overtime hours.

### 2.3. ProQoL Scale

The ProQoL Scale was developed by Stamm in 2010 [31]. It is a 30‐item questionnaire that measures three key dimensions: compassion satisfaction, burnout, and secondary traumatic stress. Each subscale includes 10 items rated on a 5‐point Likert scale from 1 (*never*) to 5 (*always*), with scores ranging from 10 to 50 [16]. In the Burnout subscale, five items (Items 1, 4, 15, 17, and 29) are reverse‐scored [31]. Each subscale is scored separately; a total score is not calculated, and subscale scores cannot be summed [16].

Standard cutoff points classify scores for each subscale as low (≤ 22), moderate [23–41], and high (≥ 42) [13]. The Persian version of the ProQoL was validated by Ghorji et al. [34], showing acceptable reliability with Cronbach’s alpha coefficients of 0.87 for compassion satisfaction, 0.87 for burnout, and 0.74 for secondary traumatic stress. In the present study, internal consistency was also satisfactory, with a Cronbach’s alpha value of 0.86 for the total questionnaire.

### 2.4. Professional Moral Courage Scale (PMCS)

This questionnaire was initially developed by Sekerka and colleagues in 2009 to evaluate professional moral courage within organizational settings [32]. Mohammadi and colleagues subsequently translated and psychometrically validated the Persian version of this questionnaire in Iran [35]. The Persian version comprises of 15 items divided into five subscales based on the original conceptual framework: moral agency (3 items; e.g., “I am tireless and persistent in doing the right thing”), multiple values (3 items; e.g., “Before making a decision, I think about how to apply organizational and personal values”), endurance of threats (3 items; e.g., “I insist on ethical issues even if faced with social pressure”), going beyond compliance (3 items; e.g., “It is important to act beyond legal requirements, and I find ways to perform my duties with ethical actions properly”), and moral goals (3 items; e.g., “Cautious power is important to me when making decisions”) [36]. Items are rated on a 7‐point Likert scale (1 = *never true* to 7 = *always true*), yielding total scores that range from 15 to 105 [26]. The Persian version of the scale demonstrated a Cronbach’s alpha of 0.81–0.85 [35]. In the present study, the internal consistency was also satisfactory, with a Cronbach’s alpha of 0.93 for the total questionnaire.

### 2.5. Professional Autonomy Scale (DPBS)

This questionnaire, originally developed by Dempster in 1990, assesses professional autonomy among nurses and other healthcare professionals [33]. The original 30‐item instrument measures four theoretically derived dimensions of autonomy: readiness (11 items related to competence, opportunity, development, and growth), empowerment (7 items related to permission, legal rights, and freedom from constraints), actualization (9 items related to the existence and enactment of autonomy in practice), and valuation (3 items related to a sense of usefulness, value, and self‐worth) [37]. Items are rated on a 5‐point Likert scale ranging from 1 (*not at all true*) to 5 *(extremely true*). The total score, therefore, ranges from 30 to 150. Four items [8, 13, 17, 26] are reverse‐scored, consistent with the original DPBS [38]. Scores are categorized as low autonomy (30–70), moderate autonomy (70–110), and high autonomy (110–150) [39]. The Persian version of this questionnaire was first translated and psychometrically validated in Iran by Aghamohammadi et al., who reported a content validity index (CVI) of 0.95 and a content validity ratio (CVR) of 0.81 [37, 40]. In the present study, internal consistency was satisfactory, with a Cronbach’s alpha of 0.81 for the total questionnaire.

### 2.6. Data Collection

Following ethical approval, a trained researcher contacted eligible nurses in the participating hospitals, explained the study objectives and procedures, and obtained written informed consent. Questionnaires were distributed during participants’ break periods and collected at the beginning of their subsequent shift. Completion required approximately 20–30 min. Participation was voluntary, and confidentiality and anonymity were assured.

### 2.7. Data Analysis

Data were analyzed using SPSS Version 27. Descriptive statistics (mean, SD, frequency, and percentage) were computed. Normality was assessed using the Kolmogorov–Smirnov and Shapiro–Wilk tests, indicating nonnormal distributions for most variables (*p* < 0.05). Before statistical analyses, potential univariate outliers were assessed using standardized z‐scores. Observations with z‐scores exceeding ±3 from the mean were identified as potential outliers. No observations meeting this criterion required exclusion from the analysis. Therefore, nonparametric tests, including Spearman’s correlation, Mann–Whitney U, and Kruskal–Wallis tests, were applied as appropriate. For normally distributed variables, independent *t*‐tests and one‐way ANOVA were used. Multiple linear regression was conducted to identify predictors of professional autonomy and moral courage, with multicollinearity assessed using VIF and tolerance and autocorrelation assessed using the Durbin–Watson statistic. For the regression models, multivariate outliers were assessed using Mahalanobis distance alongside standardized z‐scores. Demographic and occupational variables showing significant associations in preliminary analyses were considered for inclusion in the multivariable regression models. Five observations identified as outliers were excluded from the regression analyses. The significance level was set at *p* < 0.05.

### 2.8. Ethical Considerations

The study protocol was approved by the Ethics Committee of Mazandaran University of Medical Sciences (Code: IR.MAZUMS.REC.1398.621). All procedures involving human participants complied with national and institutional ethical guidelines, as well as the 1964 Helsinki Declaration and its subsequent amendments. Necessary permission to conduct the study was obtained from the relevant authorities. Prior to data collection, a trained researcher explained the study’s purpose to all participants, emphasizing that participation was voluntary and that refusal would have no negative consequences. Written informed consent was obtained from every participant. Confidentiality and anonymity were strictly maintained throughout the study, with data access restricted solely to the research team.

## 3. Results

Out of 200 distributed questionnaires, 180 were completed and returned, yielding a response rate of 90.0%. The majority of nurses (46.1%) were between 30 and 40 years old. Most participants were female (55.0%) and held a bachelor’s degree (78.3%). Regarding marital status, the majority were married (79.4%), and most nurses were employed on a contractual basis (75.0%). In terms of shift type, 52.8% had fixed shifts, while 47.2% worked rotational shifts. The majority of participants had less than 10 years of work experience (57.8%), and 52.2% reported working fewer than 40 h of overtime per month. Detailed demographic characteristics are presented in Table 1.

**TABLE 1:** Demographic characteristics of the nurses participating in the study (*n* = 180).

Variables		Number (%)
Age (years)	< 30	64 (35.6)
	30–40	83 (46.1)
	> 40	33 (18.3)

Gender	Male	81 (45)
	Female	99 (55)

Educational level	Bachelor	141 (78.3)
	Master	39 (21.7)

Marital status	Single	37 (20.6)
	Married	143 (79.4)

Employment status	Permanent nurses	45 (25)
	Contractual nurses	135 (75)

Type of shift	Fix	95 (52.8)
	Rotational	85 (47.2)

Work experience (years)	< 10	104 (57.8)
	> 10	76 (42.2)

Overtime hours	< 40	94 (52.2)
	> 40	86 (47.8)

Underlying disease	Yes	23 (12.8)
	No	157 (87.2)

The mean total score for professional autonomy was 126.88 (SD = 13.95), with a mean item score of 4.22 out of 5. The majority of nurses (86.7%) reported high autonomy, while 13.3% reported moderate levels. Among its subscales, readiness scored highest (4.23 ± 0.61). Moral courage had a total mean of 66.82 (SD = 12.74), with a mean item score of 4.45 out of 7; moral agency scored highest (4.87 ± 1.10), and endurance of threats scored lowest (4.27 ± 1.08). For the ProQoL subscales (assessed without a total score), compassion satisfaction, burnout, and secondary traumatic stress had mean scores of 38.71 (SD = 12.25), 39.10 (SD = 10.33), and 38.01 (SD = 9.95), respectively. The majority of nurses reported high compassion satisfaction (45.0%), while moderate levels were predominant for burnout (54.4%) and secondary traumatic stress (53.9%). A complete overview is presented in Table 2.

**TABLE 2:** Mean scores of professional autonomy, professional moral courage, and professional quality of life (*n* = 180).

Variables (possible score)	The number of items	Mean (SD)	^∗^Mean score per item mean (SD)
Professional autonomy (30–150)	30	126.88 (13.95)	4.22 (0.46)
Readiness (11–55)	11	46.58 (6.78)	4.23 (0.61)
Empowerment (7–35)	7	27.72 (5.90)	3.96 (0.84)
Actualization (9–45)	9	35.17 (6.30)	3.90 (0.70)
Valuation (3–15)	3	12.36 (2.12)	4.12 (0.70)
Professional moral courage (15–105)	15	66.82 (12.74)	4.45 (0.84)
Moral agency (3–21)	3	14.62 (3.31)	4.87 (1.10)
Multiple values (3–21)	3	13.24 (3.51)	4.41 (1.17)
Endurance of threats (3–21)	3	12.82 (3.24)	4.27 (1.08)
Going beyond compliance (3–21)	3	12.92 (3.50)	4.30 (1.16)
Moral goals (3–21)	3	13.20 (3.21)	4.40 (1.07)
Professional quality of life	30	No total score is calculated	No total score is calculated
Compassion satisfaction (10–50)	10	38.71 (12.25)	3.87 (1.12)
Burnout (10–50)	10	39.10 (10.33)	3.91 (1.03)
Secondary traumatic stress (10–50)	10	38.01 (9.95)	3.80 (0.99)

^∗^Professional moral courage was measured using a 7‐point Likert scale (ranging from 1 to 7), while professional autonomy and professional quality of life were measured using a 5‐point Likert scale (ranging from 1 to 5).

Spearman’s correlation analysis revealed significant positive correlations between professional autonomy and its subscales and moral courage and its subscales (*ρ* = 0.22 to 0.83, *p* < 0.05). The strongest associations were observed between overall professional autonomy and endurance of threats (*ρ* = 0.51, *p* < 0.01) and overall moral courage (*ρ* = 0.57, *p* < 0.01). Professional autonomy also exhibited significant positive correlations with all ProQoL subscales (*ρ* = 0.22 to 0.31, *p* < 0.05), with the strongest correlation between overall professional autonomy and compassion satisfaction (*ρ* = 0.31, *p* < 0.01). Moral courage and its subscales demonstrated significant positive correlations with ProQoL subscales (*ρ* = 0.18 to 0.43, *p* < 0.05). Specifically, overall moral courage was significantly correlated with compassion satisfaction (*ρ* = 0.31, *p* < 0.01), burnout (*ρ* = 0.40, *p* < 0.01), and secondary traumatic stress (*ρ* = 0.30, *p* < 0.01). Comprehensive results are presented in Table 3.

**TABLE 3:** Spearman’s correlation between the study variables (*n* = 180).

Professional autonomy							
		Readiness	Empowerment	Actualization	Valuation	Overall professional autonomy	
Professional moral courage	Moral agency	^∗∗^ *ρ* = 0.47	^∗^ *ρ* = 0.22	^∗∗^ *ρ* = 0.22	*ρ* = ‐ 0.10	^∗∗^ *ρ* = 0.29	
	Multiple values	*ρ* = 0.10	^∗∗^ *ρ* = 0.37	^∗∗^ *ρ* = 0.37	*ρ* = 0.10	^∗∗^ *ρ* = 0.44	
	Endurance of threats	*ρ* = 0.09	^∗∗^ *ρ* = 0.80	^∗∗^ *ρ* = 0.81	*ρ* = 0.11	^∗∗^ *ρ* = 0.51	
	Going beyond compliance	*ρ* = 0.08	^∗∗^ *ρ* = 0.34	^∗∗^ *ρ* = 0.53	^∗^ *ρ* = 0.17	^∗∗^ *ρ* = 0.38	
	Moral goals	*ρ* = 0.08	^∗∗^ *ρ* = 0.37	^∗∗^ *ρ* = 0.39	^∗∗^ *ρ* = 0.26	^∗∗^ *ρ* = 0.37	
	Overall professional moral courage	^∗∗^ *ρ* = 0.24	^∗∗^ *ρ* = 0.50	^∗∗^ *ρ* = 0.62	^∗^ *ρ* = 0.17	^**∗∗**^ **ρ = 0.57**	

Professional quality of life	Compassion satisfaction	^∗∗^ *ρ* = 0.19	*ρ* = 0.13	^∗∗^ *ρ* = 0.22	^∗^ *ρ* = 0.18	^**∗∗**^ ***ρ* = 0.31**	
	Burnout	*ρ* = 0.01	^∗∗^ *ρ* = 0.29	^∗^ *ρ* = 0.19	*ρ* = 0.10	^**∗∗**^ ***ρ* = 0.22**	
	Secondary traumatic stress	*ρ* = ‐ 0.05	^∗∗^ *ρ* = 0.19	^∗∗^ *ρ* = 0.37	*ρ* = 0.10	^**∗∗**^ ***ρ* = 0.26**	

**Professional moral courage**							
		**Moral agency**	**Multiple values**	**Endurance of threats**	**Going beyond compliance**	**Moral goals**	**Overall professional moral courage**

Professional quality of life	Compassion satisfaction	^∗∗^ *ρ* = 0.24	^∗^ *ρ* = 0.18	^∗∗^ *ρ* = 0.27	^∗∗^ *ρ* = 0.20	^∗∗^ *ρ* = 0.27	^**∗∗**^ ***ρ* = 0.31**
	Burnout	*ρ* = 0.08	^∗∗^ *ρ* = 0.43	^∗∗^ *ρ* = 0.29	^∗∗^ *ρ* = 0.35	^∗∗^ *ρ* = 0.33	^**∗∗**^ ***ρ* = 0.40**
	Secondary traumatic stress	*ρ* = 0.01	^∗∗^ *ρ* = 0.20	^∗∗^ *ρ* = 0.43	^∗∗^ *ρ* = 0.29	^∗^ *ρ* = 0.16	^**∗∗**^ ***ρ* = 0.30**

*Note:* Statistically significant at *p* ≤ 0.05. Bold values are main findings of this study.

^∗^ *p* < 0.05.

^∗∗^ *p* < 0.01.

Demographic comparisons, utilizing Mann–Whitney U, Kruskal–Wallis, independent *t*‐test, and one‐way ANOVA, revealed several significant differences. Moral courage scores were significantly higher among nurses working rotational shifts (*p* < 0.01) and those without underlying diseases (*p* < 0.05). Additionally, moral courage significantly differed across age groups, with the highest scores observed in the 30–40 age group. Compassion satisfaction was significantly higher in nurses with over 10 years of work experience (*p* < 0.05). Regarding burnout, nurses working rotational shifts reported significantly higher scores compared to those with fixed shifts (*p* < 0.05). No significant differences were observed for professional autonomy or secondary traumatic stress across any demographic variables (all *p* > 0.05). Complete results are presented in Table 4.

**TABLE 4:** The relationship between demographic characteristics of participants with professional autonomy, professional quality of life, and professional moral courage (*n* = 180).

Demographic characteristics		Professional autonomy	Professional quality of life			Professional moral courage
			Compassion satisfaction	Burnout	Secondary traumatic stress	
		Mean ± SD	Mean ± SD	Mean ± SD	Mean ± SD	Mean ± SD
Age	< 30	125.64 ± 15.32	37.81 ± 12.34	37.73 ± 11.01	38.32 ± 9.83	64.02 ± 11.57
	30–40	127.86 ± 12.11	38.65 ± 12.27	40.33 ± 9.73	37.39 ± 10.77	69.42 ± 13.17
	> 40	126.79 ± 15.64	40.63 ± 12.15	38.63 ± 10.36	38.96 ± 7.97	65.69 ± 12.85
	Test result	^∗^ *H* = 0.20	^∗^ *H* = 1.72	^∗^ *H* = 2.80	^∗^ *H* = 0.28	^∗∗^ *F* = 3.49

Gender	Female	126.40 ± 14.25	38.80 ± 11.77	38.54 ± 10.46	38.92 ± 9.85	66.17 ± 12.56
	Male	127.46 ± 13.64	38.60 ± 12.88	39.77 ± 10.19	36.90 ± 10.02	67.60 ± 12.98
	Test result	^∗^ *U* = 3832	^∗^ *U* = 3916	^∗^ *U* = 3730	^∗^ *U* = 3548	^∗^ *t* = ‐ 0.74

Educational level	Bachelor	127.10 ± 14.14	38.75 ± 12.26	39.26 ± 10.03	38.36 ± 10.28	67.52 ± 12.63
	Master	126.06 ± 13.37	38.56 ± 12.34	38.48 ± 11.48	36.76 ± 8.64	64.26 ± 12.97
	Test result	^∗^ *U* = 2473	^∗^ *U* = 2747	^∗^ *U* = 2681	^∗^ *U* = 2361	^∗^ *t* = 1.41

Type of shift	Fix	125.89 ± 13.29	37.12 ± 13.28	37.76 ± 10.78	37.75 ± 9.02	62.51 ± 9.52
	Rotational	127.98 ± 14.65	40.49 ± 10.78	40.58 ± 9.65	38.30 ± 10.94	71.63 ± 14.14
	Test result	^∗^ *U* = 3742	^∗^ *U* = 3560	^∗∗^ *U* = 3322	^∗^ *U* = 3737	^∗∗∗^ *t* = ‐ 5.01

Work experience (year)	< 10	126.85 ± 13.35	37.25 ± 12.27	38.69 ± 10.79	37.05 ± 10.72	66.86 ± 11.36
	> 10	126.91 ± 14.81	40.71 ± 12.00	39.65 ± 9.71	39.32 ± 8.68	66.75 ± 14.50
	Test result	^∗^ *U* = 3863	^∗∗^ *U* = 3219	^∗^ *U* = 3868	^∗^ *U* = 3534	^∗^ *t* = 0.54

Underlying disease	Yes	123.70 ± 18.12	36.26 ± 15.54	38.00 ± 10.75	36.52 ± 12.74	60.30 ± 9.40
	No	127.34 ± 13.24	39.07 ± 11.70	39.26 ± 10.29	38.23 ± 9.50	67.77 ± 12.90
	Test result	^∗^ *U* = 1701	^∗^ *U* = 1703	^∗^ *U* = 1656	^∗^ *U* = 1753	^∗∗^ *t* = ‐ 2.67

*Note:* Acceptable *p value* is *p* < 0.05. U, Mann–Whitney U statistic; H, Kruskal–Wallis statistic; *t*, independent‐samples *t*‐test statistic; F, analysis of variance (ANOVA) statistic.

^∗^ *p* > 0.05.

^∗∗^ *p* < 0.05.

^∗∗∗^ *p* < 0.01.

Five observations were identified as multivariate outliers based on Mahalanobis distance and standardized z‐scores and were excluded from the regression analyses. Multiple linear regression analysis revealed that three moral courage subscales, including endurance of threats (*β* = 0.32, *p* = 0.001), multiple values (*β* = 0.26, *p* = 0.002), and moral agency (*β* = 0.19, *p* = 0.004), along with compassion satisfaction (*β* = 0.15, *p* = 0.032), were significant positive predictors of professional autonomy. The model was significant (*F* = 12.62, *p* < 0.001, *R* ^2^ = 0.39). No significant multicollinearity was detected (VIF = 1.17–2.33; Durbin–Watson = 1.83). Also, six variables, including actualization (*β* = 0.43, *p* < 0.001), empowerment (*β* = 0.25, *p* < 0.001), readiness (*β* = 0.23, *p* < 0.001), type of shift (rotational) (*β* = 0.25, *p* < 0.001), burnout (*β* = 0.10, *p* = 0.05), and age (*β* = 0.10, *p* = 0.048), were significant positive predictors of moral courage. The model was significant (*F* = 36.21, *p* < 0.001, *R* ^2^ = 0.60). No significant multicollinearity was detected (VIF = 1.01–1.31; Durbin–Watson = 1.73). Complete results are presented in Table 5.

**TABLE 5:** Results of multiple linear regression analysis showing predictors of professional autonomy and moral courage (*n* = 175).

Predictor variable		*B*	SE	*β*	*t*	*p* value	95% CI for *B*
Professional autonomy							
	Constant	80.51	5.98	—	13.46	> 0.001	[68.70, 92.33]
1	Endurance of threats	1.35	0.39	0.32	3.44	**>** **0.001**	[0.57, 2.13]
2	Multiple values	1.05	0.33	0.26	3.16	**0.002**	[0.39, 1.70]
3	Moral agency	0.79	0.27	0.19	2.89	**0.004**	[0.25, 1.32]
4	Compassion satisfaction	0.17	0.07	0.15	2.16	**0.032**	[0.01, 0.32]
5	Secondary traumatic stress	0.13	0.10	0.09	1.23	0.219	[−0.07, 0.33]
6	Burnout	−0.08	0.09	−0.06	−0.80	0.424	[−0.26, 0.11]
7	Going beyond compliance	−0.19	0.36	−0.05	−0.51	0.609	[−0.90, 0.52]
8	Moral goals	−0.15	0.39	−0.04	−0.38	0.702	[−0.92, 0.62]
*R* ^2^ = 0.39, Adjusted *R* ^2^ = 0.36, *F* = 12.62, *p* < 0.001, Durbin–Watson: 1.83, VIF: 1.17–2.33							

**Moral courage**							
	**Constant**	**−12.25**	**6.04**	‐	**−2.02**	**0.044**	**[−24.18, −0.32]**

1	Actualization	0.87	0.10	0.43	8.11	**> 0.001**	[0.65, 1.08]
2	Empowerment	0.54	0.12	0.25	4.52	**> 0.001**	[0.30, 0.78]
3	Readiness	0.44	0.09	0.23	4.80	**> 0.001**	[0.26, 0.62]
4	Type of shift (rotational)	6.39	1.30	0.25	2.00	**> 0.001**	[3.83, 8.95]
5	Burnout	0.13	0.65	0.10	1.98	**0.05**	[0.01, 0.25]
6	Age	1.75	0.88	0.10	1.99	0.048	[0.01, 3.49]
7	Underlying disease (no)	1.90	1.97	0.05	0.96	0.338	[−2.00, 5.80]
*R* ^2^ = 0.60, Adjusted *R* ^2^ = 0.58, *F* = 36.21, *p* < 0.001, Durbin–Watson: 1.73, VIF: 1.01–1.31							

*Note:* B, unstandardized coefficient; *β*, standardized coefficient; SE, standard error; CI, confidence interval; VIF, variance inflation factor. Confidence intervals were calculated at the 95% level using the critical t‐value of approximately 1.97. Bold values are statistical significant findings.

## 4. Discussion

This study examined the interplay between professional autonomy, moral courage, and ProQoL among emergency nurses, a population practicing at the intersection of high‐acuity clinical demands and ethically complex decision‐making.

The regression findings indicate that moral courage showed the strongest independent association with professional autonomy among emergency nurses. This finding suggests that professional autonomy is closely associated with both clinical competence and the ethical capacity to uphold professional values, tolerate personal and organizational risks, and assume responsibility for independent clinical decisions [24, 41]. This interpretation aligns with Sekerka et al.’s conceptual framework, which defines moral courage as translating ethical values into action despite adversity, and with evidence showing that morally courageous nurses are more likely to advocate for patients and accept professional accountability [32].

Compassion satisfaction was the only dimension of ProQoL that independently predicted autonomy. This suggests that positive occupational resources facilitate autonomous practice by reinforcing professional meaning and engagement, whereas psychological distress (burnout and secondary traumatic stress) alone does not explain variations in autonomy. Nurses who derive meaning and satisfaction from patient care are more likely to engage confidently in clinical decision‐making and assume professional responsibility [42]. This interpretation is consistent with the JD‐R model, which proposes that work resources primarily enhance motivation and professional functioning, whereas job demands mainly contribute to strain [12]. Taken together, these findings highlight the relevance of ethical capacity, compassion satisfaction, and organizational context when considering professional autonomy, without implying that these factors causally determine autonomy.

The regression findings for moral courage indicate that Actualization, Empowerment, and Readiness remained the strongest positive predictors of moral courage, while rotational shift work, burnout, and age also showed significant positive associations. The observed associations indicate that dimensions of professional autonomy and moral courage were closely related, although the direction and mechanisms underlying these relationships cannot be established from the present cross‐sectional data. Consistent with this interpretation, recent evidence identifies psychological empowerment, work context, age, experience, and organizational factors as important correlates of nurses’ moral courage [28, 43].

Interestingly, burnout and secondary traumatic stress were positively associated with moral courage and professional autonomy. These associations should be interpreted as a pattern of complex co‐occurrence rather than as evidence of a uniformly beneficial or harmful role of moral courage or autonomy. In demanding emergency settings, ethical engagement, autonomous decision‐making, and psychological strain may occur simultaneously, as nurses who advocate for patients and confront ethical concerns may also face greater exposure to emotionally and morally demanding situations. Recent evidence similarly indicates that moral challenges, burnout, and secondary traumatic stress can coexist among emergency nurses, particularly in the presence of organizational dysfunction or inadequate leadership [19, 44, 45].

Thus, the observed associations should not be interpreted causally; rather, they may reflect the simultaneous presence of professional engagement and occupational strain within challenging practice environments. Greater moral responsibility and professional autonomy may coexist with greater exposure to demanding clinical and moral situations, particularly in contexts characterized by limited managerial and structural support [46–48]. The associations of moral courage with age and rotational shifts may likewise reflect differences in clinical exposure and opportunities to encounter ethical challenges [28]. Accordingly, moral courage and professional autonomy are better understood in relation to the organizational context in which ethical action occurs, rather than solely as individual attributes. Recent evidence supports the importance of ethical climate, leadership, and organizational support in nurses’ moral courage and responses to ethical challenges.

Notably, the strong association between endurance of threats and the organizational dimensions of autonomy (empowerment and actualization) indicates a close relationship between the ability to sustain ethical action and dimensions of professional autonomy, rather than demonstrating that one produces the other. This interpretation is consistent with recent evidence identifying moral courage as a key determinant of professional nursing behaviors, while emphasizing that supportive organizational environments are essential for translating ethical intentions into autonomous clinical practice [28, 43]. Likewise, previous studies have shown that compassion satisfaction is associated with professional values, occupational engagement, and confidence in clinical decision‐making [13, 16].

Overall, these findings support a unified interpretation: professional autonomy was associated with moral courage, meaningful professional experiences, and organizational conditions, although the relative direction and mechanisms of these relationships remain uncertain.

Across the three constructs, the findings may indicate a discrepancy between perceived individual capabilities and the organizational context in which these capabilities are expressed. This pattern may indicate a discrepancy between nurses’ perceived individual capabilities and the organizational context in which those capabilities are expressed. This overarching pattern is theoretically meaningful because autonomy, moral courage, and professional well‐being are increasingly recognized as products of the interaction between individual competence and the practice environment, rather than individual attributes alone [26, 49, 50].

Within autonomy, nurses reported the highest scores for readiness, whereas empowerment and actualization remained comparatively lower. This pattern may reflect a distinction between nurses’ perceived preparedness and competence for autonomous practice and their perceived opportunity or authority to enact autonomy within the clinical environment. Readiness primarily encompasses competence, responsibility, and professional development, whereas empowerment and actualization relate more closely to permission, freedom from organizational constraints, and the practical expression of autonomy [38]. Accordingly, the relatively high overall autonomy score should not be interpreted as uniformly high autonomy across all dimensions; rather, the findings may indicate that nurses feel prepared and capable of autonomous practice while experiencing comparatively greater constraints in translating this capacity into independent decision‐making.

Recent psychometric and cross‐cultural evaluations of the DPBS similarly conclude that autonomy cannot be achieved through competence alone; rather, it depends on empowering leadership, shared decision‐making, organizational authority, and practice environments that enable nurses to exercise independent judgment in everyday care [38, 51–53]. This distinction may be particularly relevant in emergency nursing, where nurses are required to make rapid clinical judgments but may operate within hierarchical organizational structures that shape the extent to which such judgments can be independently enacted. However, because organizational constraints were not directly measured in the present study, this interpretation should be considered contextual rather than causal.

The moderate level of moral courage observed in this study (66.82/105; mean item score = 4.45/7) warrants attention given the ethically demanding nature of emergency nursing, where time‐critical decisions and patient advocacy are integral to practice. Although no clinically established threshold defines an adequate level of moral courage, the observed score appears relatively modest compared with the variability reported in Iranian nursing populations using the same Sekerka‐based instrument [36]. This variation is consistent with recent evidence indicating that moral courage reflects the interplay of individual, moral, and organizational factors rather than a stable individual attribute [43].

From a nursing management perspective, the moderate level of moral courage should, therefore, be viewed not as evidence of deficient moral courage, but as an indicator warranting attention to the practice conditions associated with ethical action. In particular, organizational support, psychological safety, and an enabling practice environment may be relevant contextual factors for nurses’ expression of moral courage in ethically challenging situations [28, 45].

Among moral courage dimensions, moral agency represented the strongest dimension, and endurance of threats received the lowest score. This pattern suggests that recognizing ethical obligations may be less challenging than sustaining ethical actions when facing criticism, conflict, or organizational repercussions. Contemporary evidence increasingly suggests that moral courage should be understood not merely as an individual moral attribute but as an organizationally supported professional competency. Recent systematic reviews and Iranian studies consistently identify inadequate managerial support, hierarchical decision‐making, fear of negative consequences, and limited professional authority as factors associated with difficulties in translating ethical intentions into courageous clinical actions [28, 50, 54]. These findings highlight the potential relevance of both organizational and individual factors when considering moral courage, rather than demonstrating the effectiveness of specific interventions.

The evidence for ProQoL reinforces this interpretation. Although nurses maintained relatively high Compassion Satisfaction, Burnout represented the most prominent dimension, indicating that the psychological demands of emergency nursing coexisted with substantial occupational strain despite the rewarding aspects of care. Continuous exposure to critically ill patients, unpredictable workloads, time pressure, and staffing shortages may contribute to this coexistence, although these factors were not directly examined in the present study. Consistent with the present findings, recent international evidence has identified emergency nurses as being at particularly high risk of secondary traumatic stress due to repeated exposure to traumatic events and high‐intensity clinical environments [55]. Likewise, Iranian studies have consistently reported the coexistence of moderate Compassion Satisfaction with considerable Burnout and Secondary Traumatic Stress, suggesting that professional fulfillment and occupational distress frequently coexist in clinical nursing practice [56, 57].

Demographic characteristics showed only limited associations with the study variables, with organizational and professional factors showing more consistent associations across the examined outcomes. Nurses working rotational shifts reported higher moral courage, which may reflect differences in clinical exposure, ethical dilemmas, and multidisciplinary interactions, although these mechanisms were not directly assessed [28, 58]. Likewise, nurses with longer professional experience reported greater compassion satisfaction, consistent with previous evidence linking clinical experience with emotional resilience, professional confidence, and meaning derived from patient care [59, 60]. In contrast, professional autonomy, burnout, and secondary traumatic Stress did not vary across demographic characteristics, while previous evidence has identified leadership quality, staffing adequacy, ethical climate, workload, and the nursing practice environment as relevant correlates of these outcomes [54, 58, 61].

### 4.1. Practical Strategies for Nursing Management

Given the strong association of Endurance of Threats with professional autonomy, nurse managers should establish psychologically safe channels for reporting ethical concerns, provide accessible managerial support during ethically challenging situations, and use structured ethical debriefing and participatory decision‐making to help nurses sustain ethical action under organizational pressure. Such supportive organizational structures and trusting managerial relationships have been associated with greater confidence and security when nurses face ethical conflicts.

Compassion satisfaction may likewise be supported through meaningful recognition, peer support, professional development, reflective team discussions, and attention to workload and staffing conditions. Together, these strategies may strengthen an ethically supportive practice environment while sustaining nurses’ professional meaning and engagement [62].

### 4.2. Research Strengths

This study simultaneously examined professional autonomy, moral courage, and ProQoL among emergency nurses. The use of correlation and regression analyses identified key factors associated with autonomy and provided context‐specific evidence from Iranian emergency departments, addressing a gap in research on these interrelated constructs in high‐acuity nursing settings.

### 4.3. Research Limitations

The cross‐sectional design limits causal inference and prevents the establishment of the temporal direction of the observed associations; therefore, longitudinal studies are needed to clarify these relationships. In addition, reliance on self‐reported measures may have introduced common‐method and response biases, potentially affecting the observed associations among the study variables. Finally, organizational factors such as ethical climate, leadership styles, staffing adequacy, and organizational support were not assessed and may represent important sources of unmeasured confounding. Future longitudinal and multilevel studies incorporating these organizational characteristics and objective or multisource measures are warranted to provide a more comprehensive understanding of the factors associated with professional autonomy, moral courage, and ProQoL.

## 5. Conclusions

Professional autonomy among emergency nurses was strongly associated with moral courage, while compassion satisfaction provided an additional positive contribution. These findings suggest that autonomous nursing practice extends beyond clinical competence to encompass nurses’ capacity to uphold ethical values and derive meaning from patient care. However, the apparent gap between nurses’ individual preparedness and their opportunities to exercise autonomy highlights the importance of the practice environment in enabling these capacities. Nursing managers should, therefore, foster ethically supportive, empowering, and psychologically safe work environments that strengthen moral courage, sustain compassion satisfaction, and enable nurses to translate their professional capabilities into autonomous practice and high‐quality patient care.

## Author Contributions

Conceptualization: Vida Shafipour and Tahereh Yaghoubi. Data curation: Somayeh Ghorbani. Formal analysis: Mohammad Javad Ahmadzadeh‐zeidi and Seyednouraddin Mousavinasab. Writing–original draft: Mohammad Javad Ahmadzadeh‐zeidi and Vida Shafipour.

## Funding

This research did not receive any specific grant from funding agencies in the public, commercial, or not‐for‐profit sectors.

## Disclosure

All authors have read and approved the final version of the manuscript.

## Ethics Statement

The study protocol was reviewed and approved by the Ethics Committee of Mazandaran University of Medical Sciences (IR.MAZUMS.REC.1398.621). The study was conducted in accordance with institutional and national ethical standards and the principles of the Declaration of Helsinki and its subsequent amendments. All necessary permissions were obtained from the relevant hospital authorities before data collection. Before participation, all nurses were informed about the study objectives, the voluntary nature of participation, and their right to withdraw at any time without consequences. Written informed consent was obtained from all participants. Confidentiality and anonymity were maintained throughout the study, and all data were securely stored and accessed only by the research team for research purposes.

## Consent

Please see the Ethics Statement.

## Conflicts of Interest

The authors declare no conflicts of interest.

## Data Availability

The datasets are available from the corresponding author on reasonable request.

## References

[1] Weigl M. and Schneider A. , Associations of Work Characteristics, Employee Strain and Self-Perceived Quality of Care in Emergency Departments: A Cross-Sectional Study, International Emergency Nursing. (2017) 30, 20–24, 10.1016/j.ienj.2016.07.002. 27524107

[2] Amberson T. and Quarry C. , Understanding Traumatic Stress in Emergency Nurses: A Systematic Review, Journal of Advanced Nursing. (2025) 81, no. 10, 6261–6280, 10.1111/jan.16809. 39991964

[3] Gorick H. , McGee M. , Wilson G. et al., Understanding Triage Assessment of Acuity by Emergency Nurses at Initial Adult Patient Presentation: A Qualitative Systematic Review, International Emergency Nursing. (2023) 71, 10.1016/j.ienj.2023.101334. 37716173

[4] Labrague L. J. , McEnroe‐Petitte D. M. , and Tsaras K. , Predictors and Outcomes of Nurse Professional Autonomy: A Cross‐Sectional Study, International Journal of Nursing Practice. (2019) 25, no. 1, 10.1111/ijn.12711. 30426592

[5] Mrayyan M. T. , Abu Khait A. , Rababa M. et al., Professional Autonomy in Nursing: A Concept Analysis, Sage Open. (2024) 14, no. 4, 10.1177/21582440241302129.

[6] Alruwaili M. M. and Abuadas F. H. , Professional Autonomy Among Nurses in Saudi Arabian Critical Care Units, BMC Nursing. (2023) 22, no. 1, 10.1186/s12912-023-01390-x. PMC10308715 37386470

[7] Esmailpour M. A. , Shafipour V. , Shafipour L. , and Jafari H. , Factors Associated with Nurses’ Professional Autonomy: a Narrative Review, Current Psychosomatic Research. (2024) 2, no. 3, 163–172, 10.32598/cpr.2.3.238.1.

[8] Ominyi J. , Agom D. A. , and Beryl N. A. , Nursing Autonomy and Evidence‐Based Practice in Acute Care: Navigating Power and Promoting Collaboration, Nursing Forum. (2025) 2025, no. 1, 10.1155/nuf/9343933.

[9] Ahmadzadeh-Zeidi M. J. , Rooddehghan Z. , and Haghani S. , The Relationship Between Work-Family Conflict and Nurses’ Professional Commitment in Iran, 2024: A Cross-Sectional Study, Nursing and Midwifery Studies. (2025) 14, no. 4, 275–283.

[10] Balasi L. R. , Elahi N. , Ebadi A. , Hazrati M. , and Jahani S. , Barriers and Facilitators of Professional Autonomy of Clinical Nurses in Iran: A Qualitative Study, Indian journal of medical ethics. (2023) 8, no. 1, 24–31, 10.20529/ijme.2022.027. 35699278

[11] Malloy T. and Penprase B. , Nursing Leadership Style and Psychosocial Work Environment, Journal of Nursing Management. (2010) 18, no. 6, 715–725, 10.1111/j.1365-2834.2010.01094.x. 20840366

[12] Bakker A. B. and Demerouti E. , The Job Demands‐Resources Model: State of the Art, Journal of Managerial Psychology. (2007) 22, no. 3, 309–328, 10.1108/02683940710733115.

[13] Tehranineshat B. , Torabizadeh C. , and Bijani M. , A Study of the Relationship Between Professional Values and Ethical Climate and Nurses’ Professional Quality of Life in Iran, International journal of nursing sciences. (2020) 7, no. 3, 313–319, 10.1016/j.ijnss.2020.06.001. PMC7424154 32817854

[14] Galanis P. , Moisoglou I. , Katsiroumpa A. et al., Increased Job Burnout and Reduced Job Satisfaction for Nurses Compared to Other Healthcare Workers After the COVID-19 Pandemic, Nursing Reports. (2023) 13, no. 3, 1090–1100, 10.3390/nursrep13030095. PMC10443294 37606463

[15] Xie W. , Chen L. , Feng F. et al., The Prevalence of Compassion Satisfaction and Compassion Fatigue Among Nurses: A Systematic Review and Meta-Analysis, International Journal of Nursing Studies. (2021) 120, 10.1016/j.ijnurstu.2021.103973. 34102372

[16] Dilmaghani R. B. , Armoon B. , and Moghaddam L. F. , Work-Family Conflict and the Professional Quality of Life and Their Sociodemographic Characteristics Among Nurses: A Cross-sectional Study in Tehran, Iran, BMC Nursing. (2022) 21, no. 1, 10.1186/s12912-022-01069-9. PMC9624043 36316741

[17] Ness M. M. , Saylor J. , DiFusco L. A. , and Evans K. , Leadership, Professional Quality of Life and Moral Distress During COVID‐19: A Mixed‐Methods Approach, Journal of Nursing Management. (2021) 29, no. 8, 2412–2422, 10.1111/jonm.13421. PMC8420482 34254387

[18] de Wijn A. N. and van der Doef M. P. , Patient-Related Stressful Situations and stress-related Outcomes in Emergency Nurses: A Cross-Sectional Study on the Role of Work Factors and Recovery During Leisure Time, International Journal of Nursing Studies. (2020) 107, 10.1016/j.ijnurstu.2020.103579. 32446016

[19] Salimi S. , Pakpour V. , Rahmani A. , Wilson M. , and Feizollahzadeh H. , Compassion Satisfaction, Burnout, and Secondary Traumatic Stress Among Critical Care Nurses in Iran, Journal of Transcultural Nursing. (2020) 31, no. 1, 59–66, 10.1177/1043659619838876. 30957715

[20] Tang L. , Wang F. , and Tang T. , Exploring the Relationship Between Family Care, Organizational Support, and Resilience on the Professional Quality of Life Among Emergency Nurses: A Cross-Sectional Study, International Emergency Nursing. (2024) 72, 10.1016/j.ienj.2023.101399. 38198948

[21] Aquino J. , Crilly J. , and Ranse K. , The End-of-Life Care Practices of Emergency Care Nurses and the Factors that Influence These Practices: An Integrative Review, International Emergency Nursing. (2022) 63, 10.1016/j.ienj.2022.101168. 35584560

[22] Jiménez-Herrera M. F. and Axelsson C. , Some Ethical Conflicts in Emergency Care, Nursing Ethics. (2015) 22, no. 5, 548–560, 10.1177/0969733014549880. 25335919

[23] Khoshmehr Z. , Barkhordari-Sharifabad M. , Nasiriani K. , and Fallahzadeh H. , Moral Courage and Psychological Empowerment Among Nurses, BMC Nursing. (2020) 19, no. 1, 10.1186/s12912-020-00435-9. PMC7247179 32489315

[24] Hakimi H. , Mousazadeh N. , Sharif-Nia H. , Nazari R. , and Dehghani M. , The Predictive Factors of Moral Courage Among Hospital Nurses, Philosophy, Ethics, and Humanities in Medicine. (2023) 18, no. 1, 10.1186/s13010-023-00141-9. PMC10583343 37848922

[25] Li M. , Sasse J. , and Baumert A. , Standing up Against Moral Transgressions: An Integrative Perspective on the Socio-Psychological Antecedents and Barriers to Moral Courage, Handbook of Ethics Social Psychology. (2025) 189–207.

[26] Kashani M. , Bozorgzad P. , Masror Roudsary D. et al., The Relationship Between Moral Courage and Providing Safe Care in Nurses: A Cross-Sectional Study, Journal of Education and Health Promotion. (2023) 12, no. 1, 10.4103/jehp.jehp_977_22. PMC10743858 38144009

[27] Mollaei M. , Metanat F. , Javazm A. R. , and Motie M. , Exploring the Foundations and Influences of Nurses’Moral Courage: A Scoping Review, BMC Medical Ethics. (2025) 26, no. 1, 10.1186/s12910-025-01205-5. PMC11971872 40186242

[28] Li H. , Guo J. , Ren Z. et al., Moral Courage Level of Nurses: A Systematic Review and Meta-Analysis, BMC Nursing. (2024) 23, no. 1, 10.1186/s12912-024-02082-w. PMC11295526 39090605

[29] Bordbar S. , Bahmaei J. , Rad H. F. , and Yusefi A. R. , Investigate the State of Critical Thinking and Its Impact on Moral Courage and Moral Sensitivity; Evidence from Nurses’ Perspective, BMC Nursing. (2024) 23, no. 1, 10.1186/s12912-024-02496-6. PMC11558967 39533267

[30] Namadi F. , Shahbaz A. , and Jasemi M. , Nurses’ Lived Experiences of Moral Courage Inhibitors: A Qualitative Descriptive Study, SAGE Open Nursing. (2023) 9, 10.1177/23779608231157326. PMC9944332 36844423

[31] Stamm B. , The Concise Manual for the Professional Quality of Life Scale, 2010.

[32] Sekerka L. E. , Bagozzi R. P. , and Charnigo R. , Facing Ethical Challenges in the Workplace: Conceptualizing and Measuring Professional Moral Courage, Journal of Business Ethics. (2009) 89, no. 4, 565–579, 10.1007/s10551-008-0017-5.

[33] Smith D. D. N. Sc M. , Autonomy in Practice: Conceptualization, Construction, and Psychometric Evaluation of an Empirical Instrument [Disssertations], 1990, University, of San Diego.

[34] Ghorji M. , Keshavarz Z. , Ebadi A. , and Nasiri M. , Persian Translation and Psychometric Properties of Professional Quality of Life Scale (Proqol) for Health Care Providers, Journal of Mazandaran University of Medical Sciences. (2018) 28, no. 163, 93–106.

[35] Mohammadi S. , Borhani F. , and Roshanzadeh M. , Relationship Between Moral Distress and Moral Courage in Nurses, Iranian Journal of Medical Ethics History of Medicine. (2014) 7, no. 3, 26–35. PMC4263391 25512824

[36] Safarpour H. , Ghazanfarabadi M. , Varasteh S. , Bazyar J. , Fuladvandi M. , and Malekyan L. , The Association Between Moral Distress and Moral Courage in Nurses: A Cross-Sectional Study in Iran, Iranian Journal of Nursing and Midwifery Research. (2020) 25, no. 6, 533–538, 10.4103/ijnmr.ijnmr_156_19. PMC7968592 33747844

[37] Asl R. G. , Taghinejad R. , Parizad N. , and Jasemi M. , The Relationship Between Professional Autonomy and Job Stress Among Intensive Care Unit Nurses: A Descriptive Correlational Study, Iranian Journal of Nursing and Midwifery Research. (2022) 27, no. 2, 119–124, 10.4103/ijnmr.ijnmr_375_20. PMC8997176 35419265

[38] Pursio K. , Kankkunen P. , and Kvist T. , Professional Autonomy Among Registered Nurses-Validation of the Translation of the Dempster Practice Behaviour Scale and Survey Results, Nursing Open. (2024) 11, no. 5, 10.1002/nop2.2185. PMC11125569 38787920

[39] Al-Sbehat A. M. , Eshah N. F. , Rayan A. H. , Abu Raddaha A. H. , Al Omari O. , and Alhalaiqa F. , The Association Between Intensive Care Unit Nurses’ Perception of Nursing Professional Autonomy and Nursing Professionalism: A Descriptive Correlational Design, SAGE Open Nursing. (2024) 10, 1–12, 10.1177/23779608241305720. PMC11660053 39711851

[40] Aghamohammadi D. , Dadkhah B. , and Aghamohammadi M. J. I.-r , Official Publication of Indian Society of Critical Care Medicine, Nurse-Physician Collaboration and the Professional Autonomy of Intensive Care Units Nurses. (2019) 23, no. 4. 10.5005/jp-journals-10071-23149 PMC6521826 31130789

[41] Pakizekho S. and Barkhordari-Sharifabad M. , The Relationship Between Ethical Leadership, Conscientiousness, and Moral Courage from Nurses’ Perspective, BMC Nursing. (2022) 21, no. 1, 10.1186/s12912-022-00941-y. PMC9229516 35751063

[42] Pajakoski E. , Rannikko S. , Leino-Kilpi H. , and Numminen O. , Moral Courage in Nursing-An Integrative Literature Review, Nursing and Health Sciences. (2021) 23, no. 3, 570–585, 10.1111/nhs.12805. 33389792

[43] Abdollahi R. , Ghasemianrad M. , Hosseinian-Far A. , Rasoulpoor S. , Salari N. , and Mohammadi M. , Nurses’ Moral Courage and Related Factors: A Systematic Review, Applied Nursing Research. (2024) 75, 10.1016/j.apnr.2024.151768. 38490799

[44] Wolf L. A. , Individual and Systemic Etiologies and Interventions to Address Moral Distress, Moral Crescendo, Secondary Traumatic Stress, and Burnout in Emergency Nurses, Nursing Clinics of North America. (2026) 61, no. 2, 225–234, 10.1016/j.cnur.2025.09.008. 42067241

[45] Wolf L. A. and Noblewolf H. S. , Moral Courage of Emergency Nurses in Care-Limited Environments: A Mixed-Methods Study, Nursing Ethics. (2025) 32, no. 2, 514–529, 10.1177/09697330241265415. 39026509

[46] Alshammari M. H. and Alboliteeh M. , Moral Courage, Burnout, Professional Competence, and Compassion Fatigue Among Nurses, Nursing Ethics. (2023) 30, no. 7-8, 1068–1082, 10.1177/09697330231176032. 37192753

[47] Başoğul C. , Baysan Arabaci L. , Mutlu Satıl E. , and Büyükbayram Aslan A. , Professional Values and Professional Quality of Life Among Mental Health Nurses: A Cross-Sectional Study, Nursing and Health Sciences. (2021) 23, no. 2, 362–371, 10.1111/nhs.12811. 33433046

[48] Villagracia H. N. , Jacinto-Caspillo I. , Grande R. A. N. et al., Moral Courage and Resilience Protect ICU Nurses From Compassion Fatigue, Burnout, and Moral Distress: A Multicenter Cross-Sectional and Structural Equation Modeling Study, BMC Nursing. (2026) 25, no. 1, 10.1186/s12912-026-04312-9. PMC12888311 41535815

[49] Azizi Z. , Naghizadeh M. M. , and Bijani M. , The Relationship Between Moral Courage, Team Work, and Safe Nursing Care in Clinical Nurses: A Multicenter Cross-Sectional Study in Iran, BMC Nursing. (2024) 23, no. 1, 10.1186/s12912-024-02097-3. PMC11186086 38898506

[50] Rakhshan M. , Mousazadeh N. , Hakimi H. , and Hosseini F. A. , Iranian Nurses’ Views on Barriers to Moral Courage in Practice: A Qualitative Descriptive Study, BMC Nursing. (2021) 20, no. 1, 10.1186/s12912-021-00728-7. PMC8571867 34742288

[51] Abu A. A. Y. , Ayed A. , Toqan D. et al., The Factors Influencing Nurses’ Clinical Decision-Making in Emergency Department. Inquiry: The Journal of Health Care Organization, Provision, Financing. (2023) 60. 10.1177/00469580231152080 PMC9893345 36705018

[52] Goolsby M. J. , Pierson C. A. , and Sheer B. , Twenty-Eight Years of Experience Measuring Autonomy Using the Dempster Practice Behavior Scale, Journal of the American Association of Nurse Practitioners. (2020) 32, no. 10, 696–702, 10.1097/jxx.0000000000000408. 32384356

[53] Song Y. X. , Lu C. , and An Y. , The Effect of Emergency Department Nurses’ Core Self-Evaluations on Their Perceptions of Clinical Decision-Making: A Cross-Sectional Study, Journal of Nursing Management. (2026) 2026, no. 1, 10.1155/jonm/9948757. PMC12905006 41695549

[54] Lotfi-Bejestani S. , Atashzadeh-Shoorideh F. , Ghafouri R. , Nasiri M. , Ohnishi K. , and Ghadirian F. , Is There Any Relationship Between Nurses’ Perceived Organizational Justice, Moral Sensitivity, Moral Courage, Moral Distress and Burnout?, BMC Nursing. (2023) 22, no. 1, 10.1186/s12912-023-01536-x. PMC10559416 37803416

[55] Xu Z. , Zhao B. , Zhang Z. et al., Prevalence and Associated Factors of Secondary Traumatic Stress in Emergency Nurses: A Systematic Review and Meta-Analysis, European Journal of Psychotraumatology. (2024) 15, no. 1, 10.1080/20008066.2024.2321761. PMC10911249 38426665

[56] Farmahini Farahani M. , Jaberi K. , and Purfarzad Z. , Workplace Spirituality, Compassion Satisfaction, Burnout, and Secondary Traumatic Stress: A Cross-Sectional Study in Iranian Nurses, Perspectives in Psychiatric Care. (2023) 2023, no. 1, 10.1155/2023/7685791.

[57] Taghinezhad F. , Raiesifar A. , Taghinezhad Y. , and Mohammadi E. , Professional Quality of Life Among Iranian Clinical Nurses at the Forefront of the Fight Against COVID-19: A Multicenter Cross-Sectional Study, Iranian Journal of Nursing and Midwifery Research. (2024) 29, no. 2, 187–193, 10.4103/ijnmr.ijnmr_182_22. PMC11075913 38721240

[58] Ruixin Z. , Shan H. , Yongli T. , Chen J. , Qianzhu C. , and Xue W. , The Influence of Psychological Resilience and Nursing Practice Environment on Nurses’ Moral Courage: A Cross-Sectional Study, Nursing Open. (2024) 11, no. 4, 10.1002/nop2.2163. PMC11032114 38642075

[59] Deylampasha M. , Jafari H. , Ahmadzadeh-zeidi M. J. , Sharif-Nia H. , and Shafipour V. , Moral Distress, Self-Compassion, and Professional Quality of Life Among ICU Nurses: A Cross-Sectional Study in Northern Iran, BMC Nursing. (2026) 25, no. 1, 10.1186/s12912-026-04747-0. PMC13321888 42163207

[60] Yeşil A. and Polat Ş , Investigation of Psychological Factors Related to Compassion Fatigue, Burnout, and Compassion Satisfaction Among Nurses, BMC Nursing. (2023) 22, no. 1, 10.1186/s12912-023-01174-3. PMC9835325 36631763

[61] Maillet S. and Read E. A. , Areas of work-life, Psychological Capital and Emotional Intelligence on Compassion Fatigue and Compassion Satisfaction Among Nurses: A Cross-Sectional Study, Nursing Open. (2024) 11, no. 2, 10.1002/nop2.2098. PMC10851087 38391103

[62] Skyvell N. M. , Gadolin C. , Larsman P. , Pousette A. , and Törner M. , The Role of Perceived Organizational Support for Nurses’ Ability to Handle and Resolve Ethical Value Conflicts: A Mixed Methods Study, Journal of Advanced Nursing. (2024) 80, no. 2, 765–776, 10.1111/jan.15889. 37775477

